# *Life Science Alliance*, from the Academic Editors

**DOI:** 10.26508/lsa.201800044

**Published:** 2018-03-20

**Authors:** Julia Promisel Cooper, Florent Ginhoux, Sebastian Jessberger, Michael Overholtzer

**Affiliations:** 1 Telomere Biology Section, Laboratory of Biochemistry and Molecular Biology, National Cancer Institute, National Institutes of Health, Bethesda, MD, USA; 2 Singapore Immunology Network, Agency for Science, Technology and Research (A*STAR), Biopolis, Singapore; 3 Shanghai Institute of Immunology, Shanghai JiaoTong University School of Medicine, Shanghai, China; 4 Laboratory of Neural Plasticity, Faculties of Medicine and Science, Brain Research Institute, University of Zurich, Zurich, Switzerland; 5 Cell Biology Program, Memorial Sloan Kettering Cancer Center, New York, NY, USA

## Abstract

*Life Science Alliance* is a community-driven journal that brings together research from diverse fields. The journal is first-of-its-kind in uniting not-for-profit publishers and employing the scientific community to change the way authors can publish their work.

***Life Science Alliance* is a new not-for-profit, open access journal that represents a unique alliance between three leading publishers: EMBO Press, Rockefeller University Press, and Cold Spring Harbor Laboratory Press. This transcontinental partnership brings exciting, inclusive breadth to *Life Science Alliance*, as this new journal will enable transfer of work from nine major journals: *The EMBO Journal, EMBO Reports, Molecular Systems Biology, EMBO Molecular Medicine, Journal of Cell Biology, Journal of Experimental Medicine, Journal of General Physiology, Genes and Development,* and *Genome Research.***

## Why do we need another journal, and why this journal?

*Life Science Alliance* is a community-based journal that brings together carefully selected, rigorous research from diverse fields—from academic scientists, to academic scientists. Community-driven science has always been the spirit of the partner journals. *Life Science Alliance* streamlines the publication of valuable research referred to us from the nine partner journals or from direct submissions, through an editorial process led by academic editors and an advisory editorial board made up of our peers in research.

## We want to optimize the editorial process

Well-performed, valuable research shouldn't suffer from the ever-present request for one more major experiment, often at the insistence of the proverbial “Reviewer 3”. We joined *Life Science Alliance* to promote an incisive, constructive review process that aims to select for valuable research without demanding excessive revisions and additions. Rigorously performed research of interest to others should be published with minimal delay. Period. We aim to be part of the solution to the excessive revision cycles that have become routine within the modern research publication process. As scientists we know the frustration and loss of time and resources that come with such a lengthy process, and with moving from one journal to the next. As academic editors for *Life Science Alliance*, we aim to prevent such frustration by providing an alternative. We routinely evaluate manuscripts that are recommended to us from the partner journals, based on in-hand reviews, inviting authors to transfer their work to *Life Science Alliance* with a clear outline of what is needed for publication. We are also committed to handling direct submissions with one set of referee reports and only one round of major revision. As academic editors, we participate actively in decisions—in particular focussing on the essential referee requests.

## Effective change comes from a community-driven approach

The journal *Life Science Alliance* is first-of-its-kind in uniting not-for-profit publishers and employing the scientific community itself to change the way authors can publish their work. Our advisory editorial board includes not only established leaders but also numerous up-and-coming stars in their respective fields. To change the culture, we need to empower the whole community, prominently including junior faculty as well as postdocs, to review and select valuable research and to decide: What kind of science should we be publishing? What constitutes a fair and constructive review process? How do we improve the process? This is truly a community-driven journal. We invite input from the community and will continue to do so as *Life Science Alliance* evolves.

